# A Survey of Genotype and Resistance Patterns of Ventilator-Associated Pneumonia Organisms in ICU Patients

**Published:** 2019-03

**Authors:** Shabnam Tehrani, Vida Saffarfar, Ali Hashemi, Sara Abolghasemi

**Affiliations:** 1 Infectious Diseases and Tropical Medicine Research Center, Shahid Beheshti University of Medical Sciences, Tehran, Iran,; 2 Department of Microbiology, School of Medicine, Shahid Beheshti University of Medical Sciences, Tehran, Iran

**Keywords:** Gram-negative bacilli, ventilator-associated pneumonia, antibiotic resistance, ICU

## Abstract

**Background:**

Ventilator-Associated Pneumonia (VAP) occurs in hospitalized patients who have undergone intubation and mechanical ventilation for more than 48 hours. Patients referred to the Intensive Care Unit (ICU) are also affected by VAP due to specific conditions, especially by Gram-negative pathogens with advanced drug resistance. In this study, the pattern of antibiotic resistance of gram negative bacteria isolated from tracheal culture of VAP patients was investigated in ICU.

**Materials and Methods:**

In this cross-sectional study, tracheal samples were collected from VAP patients admitted in the hospital’s ICU from March 2017-February 2018. After isolation, bacterial isolates were identified using biochemical tests. Then, antimicrobial resistance pattern of these isolates was investigated using standard disc diffusion and E-test methods. Multiplex PCR were used to detect the *blaOXA-23*-like, *blaOXA-51*-like, *bla*OXA-24-like, and *blaOXA-58*-like genes among *Acinetobacter baumannii* (*A. baumannii*) isolates.

**Results:**

A total of 29 bacterial isolates were isolated from ICU patients, which were *A. baumannii, Pseudomonas aeruginosa (P. aeruginosa), Klebsiella pneumoniae (K. pneumoniae*) and candida spp, with prevalence of 38, 27.5, 13.8, and 20.7%, respectively. Antibiotic susceptibility test of isolates indicated that almost all isolates showed Multi-Drug Resistance (MDR) pattern. The *A. baumannii* isolates were resistant to ciprofloxacin and piperacillin-tazobactam, but ampicillin-sulbactam and colistin had better results. Ciprofloxacin, meropenem and colistin were effective against *P. aeruginosa* isolates, but other antibiotics were less effective and Colistin, Levofloxacin (LVX) and Piperacilin/Tazobactam were the best antibiotics that were effective on the isolates of *K. pneumonia*.

**Conclusion:**

According to the present study, high resistance to most antibiotics in gram negative bacilli showed that antibiotic therapy should be based on the type of bacteria isolated by tracheal culture and, as far as possible, combination therapies should be used to maximize the coverage of other possible pathogens, and antibiotic resistance in ICU.

## INTRODUCTION

Hospital-Acquired Pneumonia (HAP) is one the most common nosocomial infections and the leading cause of hospital acquired infection death with mortality rate of 33–35%. Among HAP patients, Ventricular-Associated Pneumonia (VAP) (1) has the highest mortality and morbidity rate and it is the second most common nosocomial infection especially in Intensive Care Unit (ICU). This infection occurs in 9–24% patients after at least 48 hours of mechanical ventilation (1–3).

ICU patients with newly acquired VAP in the ICU population often develop bacteremia, which is associated with a significantly higher mortality rate compared to nonbacteremic VAP (4, 5). The microbial etiology of bacteremia is related to the bacteria that colonize the respiratory tract. Type of etiologic agents and their antimicrobial susceptibility patterns are mostly dependent on region and hospital, local microbial flora awareness which are cause of VAP, and effective control methods are vital for improvements of the clinical outcomes.

The agents which cause VAP may be a part of the host’s endogenous flora or may be exogenously acquired from other patients, healthcare workers, devices, or the hospital environment. VAP can be divided into two categories: early-onset and late-onset. *Streptococcus pneumoniae*, *Haemophilus influenzae* and sometimes *Staphylococcus aureus (S. aureus)* often cause the early-onset VAP, whereas late-onset VAP is more frequently caused by Multi Drug-Resistant (MDR) *Pseudomonas aeruginosa* (*P. aeruginosa)*, Acinetobacter spp., or Methicillin-Resistant *S. aureus* (MRSA) (6).

Appropriate therapy in the beginning of infection is one of the most important factors for determination of VAP outcome. Hence, when VAP is suspected, antibiotics should be promptly administrated. Antimicrobial resistance is one of the threatening factors in hospitalized patients, and infection, mortality and morbidity are greater when caused by antimicrobial-resistance bacteria (7, 8). ICU pathogens are showing increasing antibiotic resistance, but vary in different countries and hospitals.

Inappropriate empirical antimicrobial therapy is known to adversely affect outcome in pneumonia associated with a mechanical ventilator (9). Treatment of VAP should be based on appropriate diagnosis such as microbiological examination of samples which have been obtained from respiratory tract through bronchoscopic Bronchoalveolar Lavage (BAL) (10, 11). According to other studies, the usage of BAL improves the outcome of VAP suspected patients (12). Therefore, this study aimed to evaluate the genotype and resistance patterns of VAP organisms in ICU patients in Labbafinejad Hospital.

## MATERIALS AND METHODS

### Ethical statement

The Ethics Committee of School of Medicine, Shahid Beheshti University of Medical Sciences approved this study (IR.SBMU.MSP.REC.1395.560).

### Isolation and identification

This cross sectional study was performed in ICU of Labbafinejad Hospital with the collaboration of Bacterial laboratory. Patients who were on mechanical ventilation for at least 5 days in ICU, who had Clinical Pulmonary Infection Score (CPIS) values (>6) including fever greater than 38°C, leukocytosis, oxygenation, progressive radiographic infiltrate and tracheal aspirate culture result, were included in the study. Exclusion criteria included: extrapulmonary infection sources, surgical history and any previous antibiotic therapy <48 hour before the study.

BAL fluid and endotracheal aspiration were obtained from all enrolled patients by bronchoscopy. Samples were immediately transported to the microbiology laboratory for bacteriological analysis. Standard conventional biochemical tests for identification of the isolates were performed on colonies from primary cultures. Also, the *Acinetobacter baumannii* (*A. baumannii*) strain identification was confirmed by 16S rRNA gene amplification and sequencing. In addition, the study included demographical information such as age, sex, underlying clinical condition, duration of hospitalization, and duration of intubation was noted for evaluation of underlying factors for incidence of VAP.

### Antimicrobial Susceptibility Testing

Standard disc diffusion method which is recommended by Clinical Laboratory and Standard Institute (CLSI 2016) was used to evaluate antimicrobial susceptibility of the isolated organisms. Accordingly, susceptibility of the isolates to following antibiotics: amikacin (30 μg/disk), trimethoprim/sulfamethoxazole (1.25/23.75 μg/disk), cefepime (30 μg/disk), ceftriaxone (30 μg/disk), piperacilin/tazobactam (100/10 μg), ciprofloxacin (5 mg), meropenem (10 μg/disk), ceftazidime (30 μg/disk) and ampicillin-sulbactam (10/10 μg) (Mast Co., Darmstadt, Germany) were examined. In addition, Minimum Inhibitory Concentrations (MICs) were determined by the E-test method according to the manufacturer’s guidelines for colistin against *A. baumannii*, *Klebsiella pneumoniae (K. pneumoniae)*, and *P. aeruginosa* (AB Biodisk). The MIC was read where inhibition of growth intersected the E-test strip. When small colonies grew within the zone of inhibition or a haze of growth occurred around MIC end points, the highest MIC intersect was recorded.

### PCR Amplification for *bla*oxa-23, *bla*oxa-24, *bla*oxa-51 and *bla*oxa-58 genes

*A. baumannii* isolates were incubated overnight at 36°C in LB medium and then DNA extraction was performed using GENETBIO (Cat.No.K-3000, Lot No.20140619). The sequences of primers used in present study are indicated in table 1.

**Table 1.** Primers used in different PCR assays

**Target gene**	**Primer**	***Gene***
OXA-23 like F	GATCGGATTGGAGAACCAGA	*bla oxa-23 like*
OXA-23 like R	ATTTCTGACCGCATTTCCAT	
OXA-51 like F	TAATGC TTT GAT CGG CCT TG	*bla oxa-51 like*
OXA-51 like R	TGG ATT GCA CTT CAT CTT GG	
OXA-24 like F	GGTTAGTTGGCCCCCTTAAA	*bla oxa-24 like*
OXA-24 like R	AGTTGAGCGAAAAGGGGATT	
OXA-58 like F	AAGTATTGGGGCTTGTGCTG	*bla oxa-58 like*
OXA-58 like R	CCCCTCTGCGCTCTACATAC	

The 25 µL PCR mixture contained 3 µL of bacterial DNA, 250 µM of each dNTP, 10 pM of each primer, 10 Mm of Tris-HCL, 1.5 mM of MgCl2, 30 Mm of KCL, and 1 U of Taq DNA polymerase (Cat. No. K-2012, Bioneer Company, Korea). The reactions were performed in a thermal cycler (Mastercycler Gradient, Eppendorf, Hamburg, Germany). The following thermal cycling condition was used for the PCR reaction: a 5-min hold at 94°C, followed then by 36 three-step cycles of; denaturation at 94°C for 45 seconds, annealing at 56°C for 45 seconds, and extension at 72°C for 45 seconds. The reaction was ended with a final 5 min extension at 72°C. The amplified DNA was run onto 1% agarose gel containing Ethidium Bromide, and was then visualized under Ultraviolet transilluminator. Molecular weight of the PCR product was determined and verified using a 100 bp standard ladder (Fermentas, Berlin, Germany).

Statistical analysis was performed using SPSS 21 software (SPSS Inc., Chicago, IL). A two-tailed P-value < 0.05 was considered statistically significant.

## RESULTS

In the present study, a total of 29 samples were collected from patients who were on mechanical ventilation in ICU of Labbafinejad Hospital from January 2016 up to December 2016, in Tehran. Twenty-nine isolates of suspected bacteria and fungi from the view of growth tests were investigated on the differential medium. The samples were cultured on selective and differential media. Colonies were evaluated for biochemical experiments. Of which, 53.3% were male and 46.7% were female, which is not significantly different. Mean age was 72.9 years old. In the present study, 97.7% subjects had fever during examinations. Meanwhile, an examination of radiographs in 80% of patients showed pneumonia. The CBC data also showed that 90% of patients had leukocytosis and 10% had leukopenia.

During the study, the samples were collected and transmitted to the microbiology lab for identification and antibiotic susceptibility testing. Different bacterial species were isolated. A total of 29 bacterial isolates were isolated from ICU patients, which were *A. baumannii, P. aeruginosa, K. pneumoniae* and candida spp., with prevalence of 38 (n=11), 27.5 (n=8), 13.8 (n=4), and 20.7% (n=6), respectively. The results of antimicrobial susceptibility testing of all isolates from VAP patients using E-test are shown tables 2–4.

**Table 2.** The results of antimicrobial susceptibility testing of *A.baumannii* isolates from VAP patients

**Antibiotics**	**Resistance**	**Intermediate**	**Susceptible**
	**NO (%)**	**NO (%)**	**NO (%)**
Amikacin	96.5%	0%	3.5%
Cefepime (FEP)	93%	0%	7%
Ceftriaxone (CRO)	93%	0%	7%
Ciprofloxacin (CIP)	100%	0%	0%
Piperacilin/Tazobactam (TZP)	100%	0%	0%
Trimethoprim-Sulfamethoxazole (SXT)	86%	3%	11%
Meropenem (MEM)	100%	0%	0%
Ceftazidime	96.5%	0%	3.5%
Ampicillin-Sulbactam (SAM)	27%	0%	73%
Colistin	0%	0%	100%

**Table 3.** The results of antimicrobial susceptibility testing of P. aeruginosa isolates from VAP patients

**Antibiotics**	**Resistance**	**Intermediate**	**Susceptible**
	**NO (%)**	**NO (%)**	**NO (%)**
Amikacin	50%	12.5%	37.5%
Cefepime (FEP)	87.5%	0%	12.5%
Ceftriaxone (CRO)	87.5%	0%	12.5%
Ciprofloxacin (CIP)	62.5%	0%	37.5%
Piperacilin/Tazobactam (TZP)	100%	0%	0%
Meropenem (MEM)	37.5%	12.5%	50%
Ceftazidime	87.5%	0%	12.5%
Ampicillin-Sulbactam (SAM)	100%	0%	0%
Colistin	0%	0%	100%

**Table 4.** The results of antimicrobial susceptibility testing of *K. pneumoniae* isolates from VAP patients

**Antibiotics**	**Resistance.**	**Intermediate.**	**Susceptible.**
	**NO (%)**	**NO (%)**	**NO (%)**
Amikacin	50%	25%	25%
Cefepime (FEP)	100%	0%	0%
Ceftriaxone (CRO)	100%	0%	0%
Ciprofloxacin (CIP)	50%	0%	50%
Piperacilin/Tazobactam (TZP)	25%	0%	75%
Trimethoprim-Sulfamethoxazole (SXT)	50%	25%	25%
Meropenem (MEM)	50%	0%	50%
Levofloxacin (LVX)	0%	25%	75%
Ampicillin-Sulbactam (SAM)	100%	0%	0%
Colistin	0%	0%	100%

One hundred percent of *A. baumannii* isolates were resistant to ciprofloxacin, Piperacilin/Tazobactam and Meropenem antibiotics. Antibiotic susceptibility to *A. baumannii* isolates indicates that isolates causing VAP infection have high resistance to common antibiotics.

*P*. *aeruginosa* isolates are resistant to Ampicillin-Sulbactam and Piperacilin/Tazobactam (TZP) by 100%. Afterwards ceftriaxone, Ceftazidime, Cefepime, and Ciprofloxacin have more resistance.

The isolates of *K.pneumoniae* had the highest resistance to Ampicillin-Sulbactam (SAM), Cefepime, Ceftriaxone and Amikacin. The most susceptible antibiotics were Colistin, Levofloxacin (LVX) and Piperacilin/Tazobactam.

In this study, the most commonly identified bacteria was shown to be *A. baumannii*, accounting for 36.7% of VAP cases.

### Multiplex PCR:

**Figure 1.**
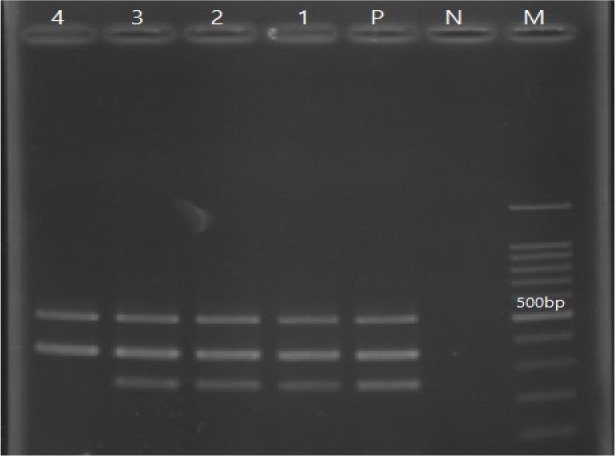
Lane M, DNA size marker; Lane N, negative control; Lane P, positive control; Lanes 1, 2, 3, and 4, positive isolates.

The *blaOXA-51*-like gene was identified in all the *A. baumannii* strains (100%) by multiplex PCR. Although the prevalence of *blaOXA-23*-like and *blaOXA-24*-like genes was 50 and 30%, respectively, the *bla*OXA-58-like gene was not detected in any of these isolates (Figure 1).

## DISCUSSION

Hospital-acquired infections have always been one of the major causes of mortality and long-term hospitalization, imposing additional economic costs on the health system and the occurrence of antibiotic resistance, which makes it difficult to control infection in hospitals especially for ICUs. Hospital pneumonia is the second most commonly reported hospital infection. According to the definition of CDC, pneumonia caused by VAP is a pneumonia that occurs after 48 hours of mechanical ventilation. Its incidence varies greatly. Tracheal intubation can increase the chance of death by VAP from 6–21 times.

Before 1972 it was thought that pathogens enter the respiratory system and cause VAP due to mechanical ventilation and its equipment, but later, it became clear that a source other than the equipment was the main cause of VAP and mechanically ventilation only provides the pathway for these pathogens. The risk factors for VAP include age, severity of illness or injury, previous hospitalization prior to admission to the ICU, mechanical ventilation time or tracheal intubation, and hospital stay in ICU, back rest, illness, underlying conditions, chronic heart disease, neurological damage, trauma, heart and internal surgery, previous use of corticosteroids, or previous antibiotic therapy (13–15).

Production of various carbapenemases, such as OXA-51, OXA-23, OXA-58, OXA-24, OXA-143 and OXA-235, has been reported as a major mechanism of carbapenem resistance in many countries, including Iran (16–20). In this study, all isolates carried *bla*_ OXA-51_ -like and 30, 50 and 0% of isolates harbored *bla*_ OXA-24_ like, *bla*_ OXA-23_ -like, and *bla*_ OXA-58_ like, respectively. OXA-23 and OXA-24 genes were previously reported to be a common mechanism of carbapenem resistance in *A*. *baumannii* worldwide (21). Mirshekar et al., showed that *bla*_ OXA-51_ -like, *bla*_ OXA-23_ -like, and *bla*_ OXA-24_ -like genes were present in 86.11 (62/72), 84.72 (61/72), and 30.55% (22/72) of the isolates, respectively, whereas *bla*_ OXA-58_ -like was not detected in any of the *A. baumannii* isolates (22). Xiao et al., found that all isolates possessed *bla*_ OXA-51_ -like gene, 95% were positive for *bla*_ OXA-_ 23-like gene and no isolates carried out both *bla*_ OXA-24_ -like and *bla*_ OXA-58_ -like genes (23). Also, Azizi et al., showed that all of the isolates carried out *bla*_ OXA-23_ -like as well as *bla*_ OXA-_ 51-like genes, while *bla*_ OXA-24_ -like was only observed in the isolates showing high MIC value to imipenem and meropenem and the *bla*_ OXA-58_ -like gene was absent in the *A. baumannii* population. Consistence to these findings, various types of *bla*_ OXA_ were identified in high level carbapenem-resistant *A. baumannii* isolates included in the present study, suggesting the role of this mechanism in resistance to carbapenems (24).

VAP diagnosis is based on clinical criteria, chest X-ray and microbiological tests. Identification of each of the potential bacterial VAPs and the exact identification of each ICU is essential to select the best antibiotic treatment and to reduce the duration of hospitalization (25, 26).

Both *Pseudomonas* and *Acinetobacter* are being increasingly reported by VAP cases. Also antibiotic resistance is quite prevalent. The type of causative pathogens might vary depending on hospital and region. In the present study, the most predominant organism responsible for infection was shown to be *A. baumannii,* accounting for 11 of VAP cases. This result is similar to other studies such as Hashemian et al. (27).

In a study in Greece that investigated Gram-negative micro-organisms in patients referred to ICU, *A*. *baumannii*, *P*. *aeruginosa* and *K*. *pneumoniae* were the most common Gram-negative bacterial agents of VAP (28). In another study, infections with *Pseudomonas aeruginosa* that were resistant to imipenem and ciprofloxacin increased significantly (29). *P*. *aeruginosa* is the second most common cause of acquired pneumonia from the hospital. Some studies have shown that *P. aeruginosa* is one of the first causes of nosocomial infections. In 2010, Jamaati et al. in a study reported a high percentage of 40% of patients with VAP (29, 30).

VAP causative pathogens vary based on case mix, methodology of sampling and local resistance patterns. According to Charles et al. study, 72.2% of VAP patients had mono microbial and 27.8% had poly microbial infection (31).

Analysis of antibiotic sensitivity pattern of organisms shows the high resistant to the commonly used drugs. In *A. baumannii*, except for ampicillin-sulbactam (SAM) and colistin, more than 90% of isolated organisms were resistant and all isolates of MDR showed a very high antibiotic resistance level among this bacterium (32). In a study which was conducted by Kazemi et al. it was shown that Acintobacter rates of resistance were equal to 46% for rifampicin, 67% for gentamicin, 100% for meropenem, 98% for piperacilin, 0% for colistin, and 96% for ceftazidim (32).

*K. pneumoniae* isolates, which contained 14.5% of the isolates, were all MDR and were resistant to a multiple antibiotic classes, and colistin, levofloxacin and Piperacilin/Tazobactam (TZP) had the highest effect on the Klebsiella species isolated from VAP patients. Yadegarynia et al., showed that about 62 *K. pneumonia* strains were isolated from clinical samples of ICU and general wards during one year. The least resistance was related to colistin (4.8%) and Amikacin (14.5%), respectively, and most resistance was related to antibiotics of ciprofloxacin (66.1%), ceftriaxone (62.9%) and gentamicin (59.7%), respectively. There was also resistance to imipenem in 38.7% of the isolates (15).

## CONCLUSION

The results of this study showed that patients referred to the ICU are more likely to develop the VAP. The bacteria isolates in the ICU at Labbafinejad Hospital in Tehran are equal to 80% gram-negative bacilli; *A. baumannii* and then *P. aeruginosa* are the most common ones*.* Precise and correct diagnosis is very important for appropriate antimicrobial treatment.
